# Exploring parental perceptions and knowledge regarding breastfeeding practices in Rajanpur, Punjab Province, Pakistan

**DOI:** 10.1186/s13006-018-0171-z

**Published:** 2018-07-03

**Authors:** Rubeena Zakar, Muhammad Zakria Zakar, Lubna Zaheer, Florian Fischer

**Affiliations:** 1 Institute of Social and Cultural Studies, University of the Punjab, Lahore, Pakistan; 2 Institute of Communication Studies, University of the Punjab, Lahore, Pakistan; 3 School of Public Health, Department of Public Health Medicine, Bielefeld University, Bielefeld, Germany

**Keywords:** Exclusive breastfeeding, Parental perceptions, Breastfeeding knowledge

## Abstract

**Background:**

Exclusive breastfeeding is significantly associated with strong infant immunity and optimal development. The importance of breastfeeding is underestimated. Parental lack of knowledge and unhealthy practices regarding breastfeeding deprive infants of their basic right to mother’s milk. In developing countries, including Pakistan, with high child mortality and malnutrition, healthy breastfeeding practices can bring positive changes in child health status. From this perspective, the present study aims to understand parents’ knowledge, attitudes and practical encounters with breastfeeding practices and the factors that prevent them from adopting such practices.

**Methods:**

A qualitative study was carried out in both rural and urban settings in Rajanpur District of Punjab Province, Pakistan. We conducted 12 focus-group discussions (FGDs) that involved 38 mothers and 40 fathers with children aged under two years who were being breastfed. A thematic content analysis of data collected through FGDs was performed manually. The themes were both inductive and deductive in nature.

**Results:**

The study found that a majority of participants believed that the first thing given to an infant after birth should not be breast milk but honey, rose flower, or goat’s milk from the hands of an elder in the family or a religious person. No cleanliness measures were practised in this regard. The participants had misconceptions about the benefits of colostrum, which frequently prevented it being given to newborns. Participants reported many factors, such as: insufficient milk syndrome (slow growth of infants due to insufficient daily breast milk intake), a mother’s high workload, lack of social support, the influence of culturally designated advisors, and the promotion and marketing strategies of infant formula companies, that undermined exclusive breastfeeding efforts and encouraged mothers to switch to infant formula.

**Conclusions:**

Culturally acceptable and integrated public health interventions are needed to improve the breastfeeding-related health literacy and practices of parents, grandparents and communities. This will ultimately reduce the high infant mortality and malnutrition rates in Pakistan.

## Background

Breastfeeding behaviours depend upon the cultural practices and perceptions that guide the actions of mothers in making decisions about the duration and frequency of breastfeeding [1, 2]. Empirical evidence demonstrates that exclusive breastfeeding for the first six months after birth contributes significantly to strong infant immunity and optimal development [1, 2]. Timely initiation of breastfeeding plays an important role in preventing infections and infant mortality [3, 4]. Breast milk is not only a primary source of nutrition for newborns but also functions as an anti-infective agent and protects breastfed infants from acute respiratory infections and diarrhoea [5]. Additionally, breastfeeding is beneficial for the mother. It is associated with elevated emotional attachment of the mother to the infant [6], reduced risk of breast and endometrial cancer [7] and increased duration of postpartum amenorrhea and consequent birth spacing [8], as well as several other health benefits such as lower risk of osteoporosis [9].

The World Health Organization (WHO) and the United Nations Children’s Fund (UNICEF) recommend the initiation of breastfeeding within the first hour after birth and exclusive breastfeeding for the first six months, especially in resource-deprived countries [10]. The WHO also advocates that breastfeeding should be continued along with complementary foods up to two years of age or beyond [10]. However, unhealthy breastfeeding practices are common in developing countries like Pakistan, where child mortality and malnutrition rates are high, and infections are widespread due to unhygienic conditions [11, 12]. The World Breastfeeding Trends Initiatives report highlights that, in Pakistan, 22% of neonatal deaths could be prevented if newborns were breastfed within an hour of birth [13]. Nevertheless, due to cultural stereotypes and false beliefs, the importance of breastfeeding is underestimated, and infants remain deprived of their basic right to breast milk, leaving them vulnerable to infections and, thus, increasing the burden of disease [3–5]. One can gauge the situation from the fact that, in the Punjab in 2013, only 10.6% of mothers breastfed their newborns within one hour of birth and only 17% of children were exclusively breastfed [14].

There are a number of cultural practices that hinder optimal breastfeeding practices, such as offering a pre-lacteal feed. A significant percentage (74%) of mothers offer a pre-lacteal feed to their newborn [14]. Because of pre-lacteal feeding, an infant is deprived of the protective benefits of colostrum. Extensive research has proved that alternative feeding increases the risk of diarrhoea and acute respiratory-tract infections [15–17], as well as poor breastfeeding outcomes, such as delayed initiation and early cessation of breastfeeding [18, 19].

Although scholars have conducted extensive quantitative studies on exclusive breastfeeding and child and maternal health, these studies have not explored the phenomenon in detail and merely provide numerical associations of one variable with another [11, 20–22]. In contrast, very few qualitative studies have been conducted to analyse the knowledge and behaviour of parents around breastfeeding practices and its benefits for the mother and child, particularly within the context of Pakistan [23, 24]. This study aims to explore the knowledge, attitudes and practical encounters of parents (both mothers and fathers) concerning exclusive breastfeeding and colostrum and their overall breastfeeding practices, and the factors that prevent them from adopting such practices. We attempted to understand the influence of cultural and spiritual beliefs on these practices, and the benefits of breastfeeding for both infant and mother. Thus, through understanding the perspective of parents and healthcare professionals – specifically public health experts and healthcare managers – we can create awareness about optimal breastfeeding practices through campaigns and other health-promotion avenues, in a manner geared specifically towards parents; possibly saving lives and raising health standards.

## Methods

### Study design

**Fig. 1:**
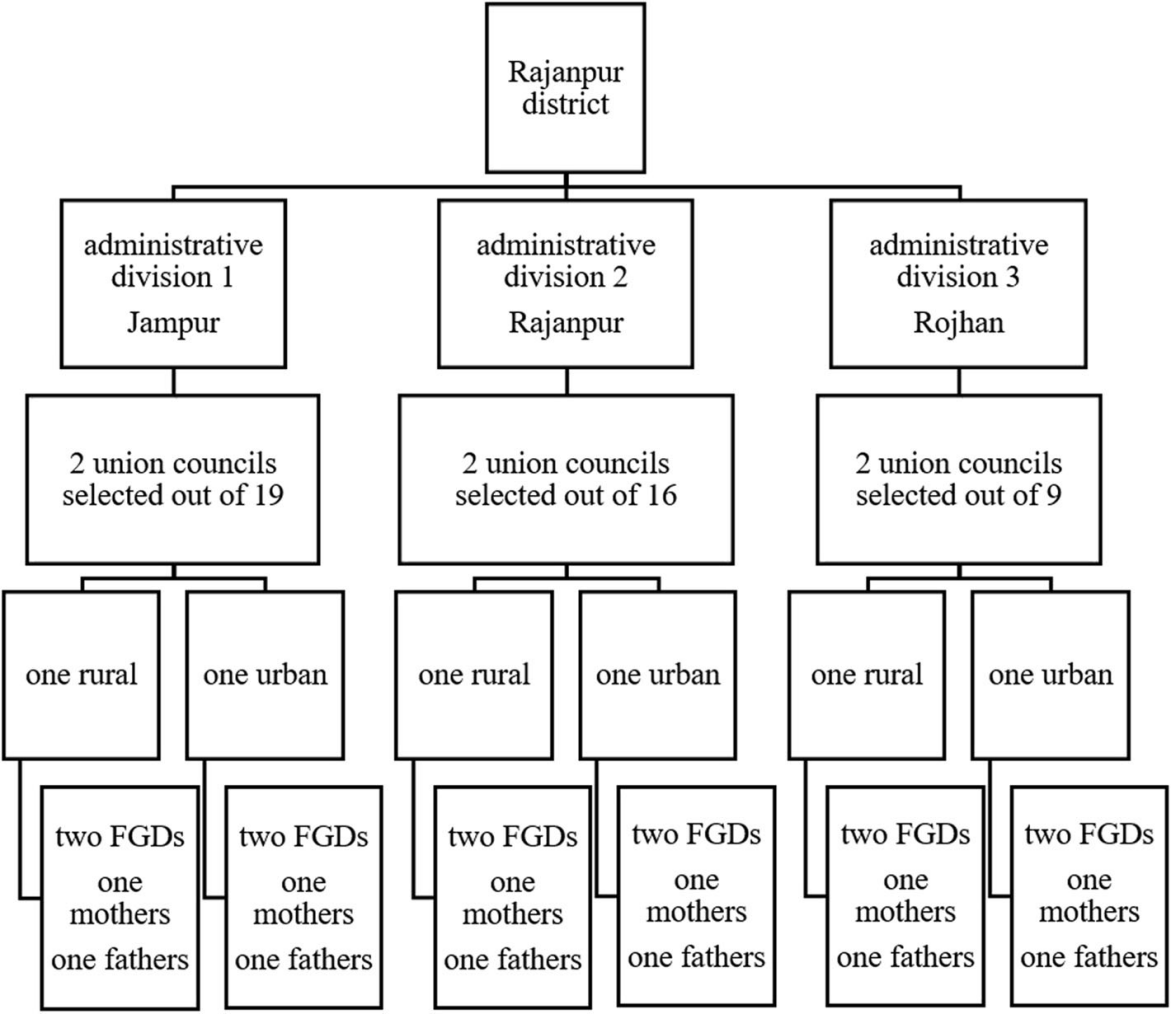
Sample selection in research setting for FGDs

The study was based on a qualitative research design. Data were collected through 12 focus-group discussions (FGDs) with mothers and fathers of children aged under two years in both rural and urban settings in Rajanpur District of Punjab Province, Pakistan. Rajanpur is situated in southern Punjab and has three *Tehsils* (administrative divisions) and 44 union councils (local government bodies). It has one of the poorest socio-economic indicators related to maternal and child health among all the districts of Punjab Province [14]. Rajanpur was selected as our study setting due to our interest in understanding the contextual factors behind child mortality and poor maternal and child health. Despite the traditional socio-economic environment, people are not practising exclusive and optimal breastfeeding, which can overcome deficiencies in child nutrition. Although about 98% of mothers breastfeed their children, very few of them (14%) initiate timely breastfeeding or practise exclusive breastfeeding [14]. Figure 1 presents the summary of union council and participant selection for the FGDs.

A purposive sampling technique was used to select mothers who had children aged under two years and were currently breastfeeding or trying to breastfeed their child. The sample was restricted in terms of the child’s age in order to minimise recall bias. Mothers who had a child with a severe illness or feeding problems (such as cleft lip or palate) were not included in the study. The data were collected from six union councils (two union councils from each *Tehsil*). In total, 12 FGDs were conducted with 38 mothers and 40 fathers (six in each category, out of which three were in urban and three in rural areas). The participants did not belong to the same families and were not couples. There were six or seven participants in each FGD. Table 1 presents an overview of the profile characteristics of participants recruited for FGDs.

**Table 1:** Profile characteristics of participants

Variables	Mothers (*n* = 38)	Fathers (*n* = 40)
Age (in years)		
Less than 25	17	9
25 and above	21	31
Number of children		
Less than 5	23	19
5 and above	15	21
Age of child (in years)		
Less than 1 year	24	27
1–2 years	14	13
Educational attainment		
No formal schooling	11	5
1–5 years of schooling	13	9
6–10 years of schooling	9	17
Above 10 years of schooling	4	9
Family monthly income (in PKR^a^)		
< 20,000	12	13
20,000–40,000	19	22
> 40,000	7	5
Family system		
Joint family	33	32
Nuclear family	5	8

^a^*1 PKR* = 107 USD

All the participants were recruited with the help of lady health workers (LHWs). LHWs are female community health workers who live in the same community where they work. Each LHW is responsible for an average of 1000 people. LHWs register the population of their service area and act as liaison between the community and health facilities. They provide antenatal care, vaccinations for children under two and health-education information on hygiene and sanitation. For the recruitment of study participants, we contacted LHWs because they knew all the families in their localities. They went door to door in their localities and explained the study objectives to mothers and fathers and invited them to participate in the FGDs. LHWs provided a list of people who had agreed to participate to the researchers. The researchers contacted the fathers and mothers who had expressed an interest in participating in the study and arranged a time and venue for the meeting according to their convenience.

The study was conducted in 2013 over a four-month period in a community setting. Eleven FGDs were conducted at LHWs’ offices, and one FGD with fathers was arranged at the house of one of the participants. The six FGDs with mothers were arranged for 10:00 am because the mothers were relatively free at that time while their older children were at school. All of the six FGDs with fathers were organised on Sundays (as this is their day off and fathers could spare enough time for the meeting). The participants were served tea and refreshments before the interview to create a friendly environment. The FGDs with mothers and fathers were conducted by female and male interviewers respectively, usually in the participants’ national language (Urdu). The interviewers were experienced in qualitative interviewing and were trained in the FGD guide before going into the field. In some cases, when participants were not comfortable with Urdu, interviews were conducted in their local language (i.e. Siraiki or Punjabi). The local LHWs were also present during the FGDs to facilitate the sessions as they had good rapport with all the participants, being members of the same community. Moreover, the presence of LHWs did not influence the content discussed in the sessions because they acted as moderators and translated certain parts of messages for researchers and participants if required. Each FGD lasted for between 90 and 120 min.

Before the start of the FGDs, all participants were informed about their voluntary participation, the confidentiality of recorded data and anonymity. Written informed consent was provided by all participants. For those participants who could not read and write, the interviewer read the consent form aloud and took their thumb impression instead of a signature. All the FGDs were audio recorded with the permission of the participants; notes were also taken during the discussions. After each discussion, the researchers discussed the topics with each other and with the LHWs to clarify the concepts and their meanings.

The study used an FGD guide to collect data from mothers and fathers. The guide was developed on the basis of an extensive review of the literature on the topic and consisted mainly of deductive themes. Also, we held informal discussions with lactating mothers and with fathers, as well as other stakeholders such as mothers-in-law, doctors, midwives, LHWs and elders in the community to incorporate contextual elements into the FGD guide. The guide was pilot tested with a group of five breastfeeding mothers before the commencement of the FGDs. The guide consisted of topics such as: the first feed given to the child after birth, the use of and misconceptions about colostrum, the timing and frequency of breastfeeding, the benefits of breastfeeding for both child and mother, perceptions about partner support, and problems related to breastfeeding. Ethical clearance for the study protocols was obtained from the Institutional Review Board, University of the Punjab, Pakistan. Throughout the research process, we assured the confidentiality and anonymity of data. The data transcripts were shredded after use in data analysis.

### Data analysis

A thematic content analysis was performed manually to analyse the data. All audio-recorded FGDs were transcribed verbatim in Urdu. The transcripts were then translated into English. Both deductive and inductive reasoning were applied in the data analysis [25]. The transcripts were read several times by the authors. A cross-check of the translations was performed to address the possibility of detracting from the contextual meaning of response statements. The field notes taken by the researchers during FGDs were also considered in order to aid the data analysis. Firstly, the initial codes were highlighted, first independently and then in joint sessions involving all the authors. A listing of all the codes was prepared. Response statements referring to the same theme were extracted and written out separately. In the second stage, the groupings of common themes/codes referring to broader categories were identified. Afterwards, thematic categories were coded to reveal the patterns and interplay of categories. The researchers moved back and forth in the transcribed data and searched for similar occurrences or repeating ideas about the same phenomenon. The refining process continued until all the instances of contradictions and similarities had been explained. Finally, the findings were presented as theoretical constructs to explain the phenomenon being studied [26]. To maintain the credibility of the responses, all the authors discussed the findings. Multiple researchers performed thematic content analysis and the initial findings were shared with the LHWs and participants to obtain their comments on whether the research findings and interpretations reflected the meanings intended and related to their personal experiences. This allowed the participants to provide corrections for errors as well as clarifications.

## Results

The areas which were discussed with mothers and fathers were related to parents’ breastfeeding knowledge and their practices. Particularly, information was sought on the use of colostrum, the exclusivity breastfeeding and the benefits of breastfeeding for both mother and infant. During the course of interviews, we tried to understand how different cultural practices can influence the breastfeeding practices of parents. Thirteen main themes were identified during the data analysis, which are presented in detail in the following section.

### Infant’s first given feed

The data analysis revealed that most of the participating mothers and fathers practised feeding a child with *ghurati*. This refers to the first food in the form of sweet things given to the newborn from the hands of some elderly and pious person in the family immediately after birth. This is done with the understanding that the child will grow up with the qualities of the person who gave the first food. These things might be honey, sugar or *Arq* [extract of rose flower], which are called *ghurati* in their native language*.* Almost all the respondents strongly believed in *ghurati* as it has a cultural and ritual significance. Some of the respondents considered it a religious obligation, so the act was religiously sanctified, and the validity of the action was rarely questioned. The cleanliness of the hands giving this food was not considered at all in this context.

### Misconceptions about colostrum and alternatives to breastfeeding

About half of the mothers (16 of 38) and fathers (20 of 40) believed that breast milk produced during the last month of pregnancy and soon after delivery (colostrum) is not suitable for a small baby. They perceived colostrum as “too heavy” [in direct translation from local language] for the newborn and believed that it cannot be digested by the infant’s fragile digestive system. Some mothers viewed colostrum as “decayed material” containing germs and other impurities. They believed that it became decayed due to its long stay in the breast during pregnancy. Therefore, discarding colostrum to clean the breast was considered a necessary and wise step.

Since the colostrum was perceived as not good for the baby, goat’s or sheep’s milk was given for the first two or three days. Mothers considered goat’s milk to be very light and easily digestible as it is relatively “thin and light” compared to the milk of a buffalo or cow. Sometimes *Sonf* [a locally produced herb which is perceived to have a good effect on digestion] was also boiled with goat’s milk and the extract was given to the baby. There was a belief among mothers that during the process of birthing, babies can ingest various smelly and dirty fluids. Thus, it is considered necessary to give goat’s milk to clean and drain that fluid out of the stomach. In order to clean the abdomen of the baby, *Arqs* [rose water] is exclusively given to newborns for one or two days for “cleaning purposes” and then after two days breast milk is started. Mothers reported that this behaviour is usually followed by most mothers as it has been practised for generations.

In addition, there is a cultural belief among mothers that milk comes three days after birth. One mother who recently gave birth to her fourth child and lives in a rural area, stated:

“When a woman delivers a child, she is in great pain; she cannot eat properly, and her body is dirty. In such a state, how could she feed her child? There may be no milk and even if there is some, it may not be healthy for the newborn. .. So it is advisable to wait for some time before feeding.” (G2, P4, M22).

### Benefits of breastfeeding for the mother

Some male participants said that they were not aware of any specific benefits of breastfeeding for a mother, but they were sure that it is a healthy activity. Almost all the mothers perceived breastfeeding as a natural activity having many benefits without any harm.

A farmer with no formal schooling stated: “It is the command of Allah; so why think about any benefit? Order is order.” (G1, P6, F32).

Overall, the discussion demonstrated several benefits, as realised by both mothers and fathers. These included the fact that breast milk is readily available round the clock, and it was considered cost-effective and to have the required nutrients to strengthen an infant. However, not a single participant reported anything about improving mothers’ dietary nutrients during lactation. There was much concern about making an infant fat, which implied that the fatness was considered to be healthy and powerful. The benefits of breastfeeding were seen by participants as being more important and advantageous for labouring-class families, who did not own a refrigerator or sophisticated heating appliances in the kitchen. A female farm worker reported that:

“Labouring-class mothers are too busy in their household and farm work and they don’t have time to properly sterilise the bottle: they don’t have fridges or other facilities. Formula milk is, therefore, not feasible for them… We earn our living with great difficulty and can only feed our child with breast milk.” (G3, P4, M28).

There was a general perception among the participants that, after discarding colostrum for a few days, the breast milk becomes healthy and easily digestible for the newborn. Mothers agreed that breastfeeding increases the intimacy between a mother and her baby and that it has a positive health impact for both the baby and the mother. Furthermore, they reported that the risk of cystic formations in the breast and many other ailments had been reduced. Some male participants were of the view that poor families could not afford to buy infant formula and other alternatives so they relied on breastfeeding for their infants. They believed that if a mother was not producing milk due to weakness or health complications, a child might grow up with deficiencies and weak immunity. In addition, breastfeeding was perceived to be a natural birth prevention mechanism. Despite enumerating many benefits of breastfeeding for mothers, some of the participants believed that these benefits could only be “materialised” if the mother got proper food and was healthy.

“If the mother is weak [referring to anaemia], living in a poor and unhealthy environment and has no access to nutritious food, she will get less benefit from breastfeeding. Rather, breastfeeding will drain her energy and make her *dhaancha* [referring to her body structure turning into skin and bones].” (G1, P2, M36).

### Benefits of breastfeeding for the infant

Invariably, all the participants considered breastfeeding extremely beneficial for the health and growth of a newborn. Some of the respondents believed that “it is a sin if the baby is deprived of breast milk”. The respondents attached some cultural notions of power/strength to breast milk. Most of the participants mentioned that breast milk provides “preventive energy” for babies; if the baby has proper breastfeeding, “hundreds of diseases and ailments can be warded off”.

One father mentioned that breast milk contains some “special and lasting power… the impact and power of breast milk lasts for 40 years.” (G6, P3, F34) Another one stated:

“Breastfeeding is good for an infant. Medical knowledge has not yet discovered and counted the benefits of this natural gift [referring to breast milk] to the infant. Our forefathers used to be healthy and powerful because they got proper breastfeeding. There was no concept of diabetes, blood pressure or ulcers because the children were exclusively breastfed then…” (G4, P1, F41).

### Socio-cultural belief system concerning breastfeeding

Some of the mothers and fathers believed that breast milk transmitted behavioural traits from mother to baby. However, linking breast milk with a mother’s religiosity and piety is a tricky proposition. The misbehaviour of a child in later stages of life are blamed on the breast milk and poor attitudes of the mother. These arguments were supported by religion:

“If a noble and God-fearing mother breastfeeds, it will make the baby noble and God fearing as well.” (G4, P2, F48).

“Breast milk has an impact on the behaviour of an infant. If the mother is deviant, sinful or of ‘loose character’ she may not be allowed to breastfeed. In such cases, cow’s milk is better.” (G3, P5, M36).

“Breastfeeding is a command of Allah. His commands are always beneficial and good for the health and well-being of mankind… The training of a child begins with breastfeeding.” (G2, P1, M29).

### Causes of bottle-feeding and non-production of breast milk

Participants were asked: why do some mothers prefer bottle-feeding instead of breast milk? The main reason stated was that mother’s milk was not available in sufficient quantity or that some mothers did not have any milk at all. It was rarely reported that a healthy mother opted to bottle-feed her child. The mothers who had undergone caesarean section also reported not breastfeeding. Nevertheless, they could not explain any relationship between caesarean section and non-production of breast milk. One mother, who had herself undergone the experience of caesarean section, said: “After the operation, the doctor prescribed strong medicine [referring to antibiotics], which dried up the milk.” Another woman believed that the food produced by excessive use of fertilisers and pesticides may cause an insufficiency or total absence of breast milk. Some mothers reported that, in the case of delivery in a private hospital, doctors prescribed and encouraged the use of expensive infant formula after a caesarean operation.

“After a caesarean operation, the mother cannot feed, as the baby is kept in a nursery. How can the mother feed the baby? The lady doctor prescribes some imported formula milk. So the baby is started feeding with bottle milk and then it gradually gets used to bottle feeding. Almost all the babies born through caesarean are bottle-fed.” (G2, P2, M32).

Negative connotations were implied for formula feeding. Mothers as well as fathers believed that rich people and fashionable women choose to formula feed their babies.

“Formula milk is *dekhawa* [snobbery] and a show of wealth and modernity: wealthy people buy expensive milk to show their wealth.” (G5, P4, F43).

We found contradictory opinions about the effects of breastfeeding on mothers’ bodies. Some women believed that breastfeeding practices could improve the feminine shape of the female body. However, others perceived that the practice de-shapes the female body. These mothers avoided breastfeeding because they believed that it would make their bodies lose shape. They further reported that some “modern women” who took more care of their health perceived that breastfeeding would weaken their bodies and would have a negative impact on their fitness. The respondents believed that mostly educated, middle-class families think in this way.

Some people were concerned about the poor quality of milk, which made the baby ill. Upon such suspicions about the quality of their milk, breastfeeding was stopped by mothers. Some people believed that in certain circumstances, due to the mother’s ailments or unknown reasons, the mother’s milk becomes poisonous; it makes the baby ill and pale [referring to anaemia], and breastfeeding should be stopped immediately. The local culture had a technique to “empirically verify” the quality of milk. One mother stated: “Sometimes the mother’s milk is poisonous. In such cases, the mother’s milk is extracted and is given to small insects. If the insect dies, it is construed that the milk is poisonous.” (G2, P7, M37) There were also other reasons that discouraged exclusive breastfeeding. Some participants stated that some mothers start infant formula as supplementary food and others use it to increase the weight of the baby. One mother said: “Mother’s milk is thin, so the baby remains thin and weak. If you want to increase the weight of the baby, you may give bottle milk.” (G6, P1, M35).

Some participants underlined certain medical conditions when breastfeeding is to be avoided. One of the male participants reported: “When the mother is suffering from tuberculosis or any other malignant disease, she should avoid breastfeeding. Because, the local culture believes, the disease could be transmitted to the baby through the breast milk.” (G3, P4, F36) A female participant shared: “If the mother is ‘too weak’ she ought to stop breastfeeding. It could be harmful to the health of the mother as well as the baby. Milk from a mother who is too weak may not be beneficial for her baby.” (G2, P5, M23) Nevertheless, other participants disagreed with this idea.

### Knowledge about exclusive breastfeeding

The data analysis revealed that mothers do not practise exclusive breastfeeding, which has positive health implications for both child and maternal health. Cultural practices were preventing mothers from doing so. The lack of health literacy and guidance about healthy breastfeeding were contributing to the poor health of children. The majority of mothers and fathers were unable to comprehend what exclusive breastfeeding was about. Irrespective of the fact that an infant needs only the mother’s milk during the first six months, people preferred alternative ways to feed their baby, which were mainly backed by non-scientific sources. Both mothers and fathers believed that keeping a child on mother’s milk for six months would not make the baby fat. The strength needed to crawl was associated with the semi-liquid feed given from the fourth month.

### Desired duration and frequency of breastfeeding

We found a common perception about the duration and frequency of breastfeeding. It was believed that the mother should breastfeed the baby as long as possible. Nevertheless, they had no clear idea about the desirable duration of breastfeeding. The women reported having breastfed for between seven months and four years. It was also reported that the duration of breastfeeding depended on factors such as: 1) the occurrence of the next pregnancy; 2) the quantity of milk produced; and 3) the quality of milk. While discussing the duration of breastfeeding, some of the male participants made reference to the Holy Quran, which stipulates a duration of two and a half years. In the light of this Quranic reference, some male participants considered it sinful not to breastfeed a baby. They considered breast milk an “inalienable right of the baby” and very important for his/her proper growth and development.

“Breastfeeding duration is not in the hands of mothers; it depends on the next pregnancy. So, it should be given until the next pregnancy or as long as milk comes.” (G3, P5, F29).

### Mothers’ diet during lactation

Most of the male participants considered that no special diet is needed during lactation under normal circumstances. They further reported that a mother only needs special food in cases when she is too weak or the production of milk is not sufficient. Female participants also believed that no special diet is required; what is normally available at home is sufficient. Nevertheless, they underlined the importance of *ghee* [clarified butter made from the milk of a buffalo or cow], and believed that a lactating mother should eat it regularly.

One mother of four children opined: “The mother should eat at least 5 kg of ghee during *Chila* [the post-partum period lasting for 40 days].” (G6, P5, M39).

However, some respondents thought that fruits and vegetables were also desirable food, but not essential. One father stated: “The mother should drink plenty of cow’s milk. If she cannot arrange milk due to poverty, then *lassi* [a sweet or savoury drink made from a yogurt or buttermilk base with water] may be the best option.” (G3, P4, F42).

It may be interesting to note that there was more stress on “what not to eat” instead of “what to eat” during the lactation period. The participants largely adhered to the local belief system of a “hot” and “cold” classification of things. Invariably, all the participants were of the view that “hot things” (e.g. eggs, chicken, beef, fish etc.) should be avoided. They stated some reasons behind this belief. One mother of four young children reported: “The intake of excessive hot food turns the breast milk hot and therefore it is difficult for the small baby to digest it.” Secondly, sour things (like all citrus fruits, such as lemons etc.) were also considered harmful for the breastfeeding mother and her baby. Hard-to-digest things (like lentils and pulses, especially *Dal channa*) were also considered harmful. They also said that mothers should not take excessive tea or spicy food.

One male participant, who taught at a local high school, stated: “A lactating mother must keep a balance in her food intake to provide healthy milk for her child. She must not eat ‘too hot’ or ‘too cold’ food.” (G3, P2, F45) In the words of one mother-in-law stated by a mother [said in informal conversation]: “A mother must not keep eating ‘like a goat’; rather she should be careful and responsible.” (G1, P5, M39).

### Lactation-related problems experienced by mothers

Various problems related to breastfeeding were discussed. The most commonly reported problem was tears on the nipples, which caused a lot of pain to the mother. One mother shared: “Due to tears on my nipple, it was virtually impossible for me to breastfeed my baby.” (G4, P2, M23) Some of the women reported the problem of breast abscesses and said that severe pain in the breast was an obstacle to breastfeeding. Most of these problems were treated with home remedies and spiritual treatments. About half of the female participants reported the problem of producing insufficient breast milk for the child. A majority of these women said that this was due to the lack of a proper diet eaten by the mother. Some women stated that, due to their extensive engagement in household and farm activities, they could not feed their babies frequently enough, and this infrequent suckling led to a reduced production of milk.

### Women’s perceptions about lactational amenorrhea

Some of the mothers considered lactational amenorrhea harmful to the health of the mother. While explaining the ill effects, one mother explained:

“If *mahwari* [referring to the menstrual cycle] is stopped, that means decayed and “bad” blood is being accumulated within the body. Definitely this would have dangerous consequences for the health of women. Sometimes it causes a constant headache or weakening eyesight, or it may cause general decay of the whole body.” (G3, P1, M34).

However, a majority of the mothers disagreed with this comment and thought that missing cycles has no ill effects on the body. One mother shared: “Missing menstrual cycles is good in two ways: First, it prevents the next pregnancy and, second, it prevents the wastage of blood.” (G1, P3, M25).

### Inter-spousal communication

There was a complex normative system regulating inter-spousal communication in the local culture. Cultural norms usually encouraged the involvement of husbands in issues related to reproductive health. When male participants were asked about their communication with their wives in matters relating to reproductive health, at the outset, the response was negative. A majority of the female participants reported that their husbands were not at all concerned with lactation and pregnancy-related things and “they ought not to be.”

One mother said: “No one would appreciate it if a man takes an interest in women-related issues, they [men] may not have proper knowledge and may offer bad advice.” (G4, P1, M29).

Another female participant explained: “The responsibility of the husband is to earn and provide necessary household things for the wife; this is enough, it is better to mind his own business.” (G6, P2, M32).

Mostly the female participants believed that a woman should first consult her mother-in-law and other elderly wise women and she may also inform her husband if there is something serious. One woman in her mid-thirties reported: “Informing the husband about women-related problems may be good but it is useless. Men in villages have no information or training about women’s health problems.” (G5, P2, M36) Some of the female participants highlighted the issue of discrimination against female babies. One mother of three girls and one son shared:

“My husband never took any interest in breastfeeding-related issues when I had female babies. He never cared about my diet during lactation. But recently, I gave birth to a baby boy. Now he takes a lot of interest and asks me about my diet.” (G5, P4, M38).

### Information system and dissemination

Participants employed different information-seeking mechanisms in rural and urban areas. The rural respondents were mostly illiterate or semi-literate and their main source of information was their elders and relatively more informed neighbours. “People discuss things with trusted people and then make decisions accordingly”, observed an illiterate female respondent. However, in urban areas the literacy rates were high and people received information from written material, the mass media – especially TV debates – and advertisements, healthcare staff and educated friends.

Participants were asked about their information-seeking and dissemination mechanisms. A majority of female (30 of 38) and male (32 of 40) participants reported interpersonal communication as the most effective way to seek and share information. It was also pointed out that group discussions were important for providing information. Participants stated that advertisements on television and the radio also provided information, but that such one-way information is usually ignored by end-users. Some of the female participants suggested that illustration-based, easy-to-understand leaflets and pamphlets could be useful to improve and update their level of information.

## Discussion

This study explored the knowledge, attitudes and practices of mothers and fathers with children under two years of age regarding their breastfeeding practices. Breastfeeding is not a matter of the awareness of mothers; rather, the behaviour is governed by a host of stakeholders, such as the mother-in-law, husband, neighbours and relatives of the in-laws, local healers and religious leaders. All act as advisors to the lactating mother and define the appropriate “quality” and “suitability” of her breast milk. The mother’s sexuality and a further pregnancy are also considered to “spoil” her breast milk, and these ideas create misconceptions that such spoiled milk can cause diarrhoea, vomiting and weakness in the child [27].

We suggest that breastfeeding needs to be viewed, not in narrow terms as a mother-child dyad, but as taking place within a wider household and community environment in which other actors and informal communication networks operate and influence such practices. Particularly, the influence of the grandmother and culturally designated advisors and caregivers play a crucial role in mothers’ decision-making about the dis/continuation of breastfeeding [28]. The data also reveals that, sometimes, discontinuing or terminating breastfeeding may not be the decision of the mother. It may be erroneously assumed to be due to her lack of awareness, but is rather precipitated by her feelings of disempowerment in the face of powerful advisors who may readily blame her for negligence or inappropriate behaviour. One needs to understand the interconnections between social relations, resources, sexuality, embodiment, power, and nurturance, which are all implicated in the challenge of feeding a newborn infant [29].

Cultural beliefs were found to be a major obstacle standing in the way of exclusive breastfeeding. We found that there was little accurate knowledge or awareness among people about exclusive breastfeeding practices. The socio-cultural patterns of breastfeeding practice are considered to be part of their religious traditions and a centuries-old, time-tested exercise [22]. After birth, breastfeeding was delayed due to various ritual and symbolic reasons. The local culture attached some sort of “honour to the first feed” and there were a number of symbolic activities connected with this honour [26]. Usually, the mother was not the one to whom this honour accrued because she was considered impure due to the very process of birthing. There was a deeply held belief that the baby may acquire behavioural traits from the person who first feeds him/her. Hence, some family patriarch (usually the grandfather or a notable religious figure) whose behaviour deserved to be emulated did the job.

Although the health and immunological benefits of colostrum are well documented, “the local culture attached different meaning and importance to this body fluid as the cultural understanding is different than the universal physiological facts” [30]. In Pakistan, as in many developing countries, there is a negative cultural construction about the nature and effects of colostrum; it is largely considered unclean and unhealthy for the newborn. This belief results in the discarding of the colostrum and a delay in breastfeeding while the newborn is given something else as a pre-lacteal feed [31]. Statistical evidence demonstrates that, in the Punjab, the prevalence of pre-lacteal feeding is 75% [32]. Due to lack of knowledge, cultural myths and traditional practices, only 17% of children under six months of age are exclusively breastfed in Pakistan [32].

Our study found many misconceptions about the introduction of colostrum. Such misconceptions are not limited to mothers in Pakistan; mothers in India [33] and Bangladesh [34] also believe that colostrum may harm the newborn because it is stored in the breast for the whole of the pregnancy. For that reason, adequate initiatives for improving knowledge about breastfeeding practices are needed. The participants had diverse ideas about the benefits of breastfeeding for both mother and newborn. Overall, the local culture considered breastfeeding a beneficial and natural activity and emphasised its continuation. However, these benefits and desirability were conditional, and strictly regulated by various cultural beliefs and local techniques to check the quality of the breast milk [31]. How these cultural beliefs intersect with medical technology, information, the market and globalisation did not fall within the scope of this research. Nevertheless, it was noted that these beliefs vary between social classes. Such beliefs also differ with respect to the level of education of the parents, their income and their level of health literacy [35].

The present study found that insufficient milk syndrome was another factor undermining exclusive breastfeeding and encouraging the use of infant formula. On the pretext of the mother having insufficient breastmilk production, a decision regarding supplementing or terminating breastfeeding was made [36]. However, “insufficient” is a subjective quantity depending on the perception of the mother or her advisors, and the normal quantity was never operationalised. Other studies have reported that the claim of having insufficient milk may be attributable to women using it as a culturally acceptable explanation for using infant formula [37]. Many studies have provided evidence that women continue to produce breast milk of adequate quality and quantity even when they themselves eat a minimal diet [38].

As in other developing countries, this research documented that mothers experience various dietary and behavioural restrictions (e.g. not to eat “hot food” or have sex during breastfeeding, etc.). Such restrictions are sometimes too stringent and harsh, so that women feel “unqualified” to produce “good milk”; and instead of giving “bad milk” to the baby, the very process of breastfeeding is terminated. Sometimes these regimented requirements and restrictions are imposed by partisan family members to advance their vested interests; for example, a mother-in-law may want to make breastfeeding difficult for the mother and then press her to engage in household work; or a husband may put restrictions on breastfeeding to enable the resumption of sexual activity, which is culturally barred during the breastfeeding period [39].

Elaborate cultural techniques to declare milk “poisonous and dirty” may be another example of the active role taken by advisors and stakeholders in the process of breastfeeding [40]. For example, it has been observed that women who question the quality of their breast milk have a strong incentive to stop breastfeeding in order to avoid their baby becoming sick. Women’s anxieties about the quality of their milk may be increased both by folk healers (who float insects in the milk) and by hospital pathology laboratories (which perform pseudo-scientific analysis for “bacteria” and “pus cells”). For these reasons, women are special targets of commodification, when their bodies are valued for their reproductive potential [41].

The results also showed that there was a lack of social support. Notwithstanding the verbal claims about the virtues of breastfeeding ranging from the commandment of Allah to the high status of breastfeeding mothers, the reality is that mothers often find little social support to continue breastfeeding. For example, their workload is not decreased, and appropriate food is not provided. Rather, sometimes, a breastfeeding mother is blamed for the poor or poisonous quality of her milk. In this situation, the mother discontinues breastfeeding for fear of being blamed for making the baby sick by providing unhealthy breast milk. The data showed that various local conceptions and beliefs about the attributes of breast milk and its alternatives, could have considerable impact on the pattern of breastfeeding [42, 43].

In Pakistan, about 50% of the rural population receives treatment from quacks and traditional healers [44]. As in other developing countries, these unsupportive and untrained individuals may undermine breastfeeding efforts [37].

Many problems were linked with breastfeeding; among others, problems of suckling, nipple pain and breast abscesses were discussed. It was noted that there are many culturally designated advisors, caregivers, community workers, and family elders who continue to provide guidance to the mother. Sometimes, this advice is contradictory and creates confusion and anxiety for mothers [45].

Previous studies have reported that, despite knowing the importance and benefits of breast milk, people think that infant formula makes the baby healthier [46]. This belief, though bio-medically not proven, may be due partly to the watery nature of breast milk and partly to the marketing strategies of infant formula companies, which present their product as being of superb quality [47].

This research has identified that various physical, social and ideological ecologies create a given cultural context, which supports or constrains decisions about breastfeeding, especially its duration and exclusivity [42]. It was noted that the mother’s physical environment and familial relations influence feeding patterns. More specifically, the mother’s household workload, living patterns, and reproductive schedule modify maternal decisions regarding the continuity of breastfeeding. In short, the overall social and institutional environment within which the mother is situated is considered an important predictor of breastfeeding behaviour.

### Limitations

A major strength of this study was the comparatively large design with 12 FGDs. In addition, the combination of a deductive and inductive approach in data analysis has led to a comprehensive assessment of topics related to parental perceptions and knowledge regarding breastfeeding practices. As for any qualitative study design, the findings may not be generalisable. However, we expect that the results provide a good picture for similar populations. Interviews were conducted by trained interviewers. Nevertheless, the use of different languages may also influence the results. In addition, it has to be noted that the focus-group discussants may have provided socially desirable answers.

## Conclusions

The breastfeeding-related health literacy of parents, grandparents and community needs to be improved to reduce the high infant mortality rates in Pakistan. This can be achieved through targeted, culturally acceptable, and integrated public health interventions at individual, community, and national levels. Such interventions include: engaging social and family decision-makers, involving men to motivate and support mothers, establishing peer-support social networks, and creating awareness through mass media about the benefits of the early and timely initiation of breastfeeding. These are imperative for promoting healthy breastfeeding practices in Pakistan. A more practical approach would be to train LHWs and midwives about the importance of child and maternal nutrition, which can be achieved through encouraging exclusive breastfeeding. Additionally, media campaigns and talk shows should be used to disseminate information about the dietary intake of mothers who are breastfeeding. Efforts should be directed towards changing the main sources of information regarding the postpartum period and breastfeeding in order to eradicate false perceptions.

## Abbreviations

FGD: Focus-group discussion
LHW: Lady Health Worker
UNICEF: United Nations Children’s Fund
USD: United States Dollar
WHO: World Health Organization
